# Renal Function Is Related to Severity of Coronary Artery Calcification in Elderly Persons: The Rotterdam Study

**DOI:** 10.1371/journal.pone.0016738

**Published:** 2011-02-02

**Authors:** Abdelilah el Barzouhi, Suzette Elias-Smale, Abbas Dehghan, Rozemarijn Vliegenthart-Proença, Matthijs Oudkerk, Albert Hofman, Jacqueline C. M. Witteman

**Affiliations:** 1 Department of Epidemiology, Erasmus Medical Center, Rotterdam, The Netherlands; 2 Department of Radiology, University Medical Center Groningen, University of Groningen, Groningen, The Netherlands; Lerner Research Institute, Cleveland Clinic, United States of America

## Abstract

**Background:**

Coronary artery calcification (CAC) has been proposed to be the underlying mechanism of the increased risk of coronary heart disease with reductions in glomerular filtration rate (GFR). Since renal function diminishes with aging we examined the association between GFR and CAC in the Rotterdam Study, a population-based study of elderly individuals.

**Methods:**

The study was performed in 1703 subjects without a history of coronary heart disease. GFR was estimated using the modification of diet in renal disease equation. We used analysis of covariance to test for mean differences in CAC between GFR tertiles.

**Results:**

The mean CAC scores in the middle and lowest GFR tertile did not significantly differ from the mean CAC score in the highest GFR tertile (geometric mean CAC score 4.1 and 4.3 vs 4.2). In a multivariable model the mean CAC score did also not differ between the GFR tertiles. As the interaction term between age and GFR was significant (P = 0.037), we divided the population in two age categories based on median age of 70 years. Below 70 years, the mean CAC scores did not differ between the GFR tertiles. Above median age, mean CAC score in the lowest GFR tertile was significantly higher than the mean CAC score in the highest tertile in a multivariable model (CAC 4.9 vs 4.5, p = 0.010).

**Conclusion:**

In this population-based study we observed that the association between CAC and GFR is modified by age. In participants at least 70 years of age, a decrease in GFR was associated with increased CAC.

## Introduction

Patients with chronic renal insufficiency appear to have a higher risk of coronary heart disease (CHD), independent of cardiovascular risk factors [1]–[10]. One of the possible underlying mechanisms is that renal dysfunction leads to acceleration of coronary artery calcification (CAC) [11], [12], detectable by Computed Tomography (CT), which is a strong and independent risk factor for CHD [13]–[15]. Studies indeed showed that renal impairment is associated with higher CAC scores in pre-dialysis and dialysis patients [12], [16]–[19].

However, in population-based studies, in which persons typically had mild to at most moderate renal dysfunction, the association between impaired renal function and CAC either was not significant or strongly attenuated after adjustment for cardiovascular risk factors [11], [20]. However, the population-based studies thus far may have had negative results because their study populations were relatively young while renal function is known to diminish with advancing age [21]–[23]. Within the Rotterdam Study, a large, prospective cohort study among elderly, we investigated whether mild to moderate renal dysfunction is related to increased amounts of coronary calcification.

## Methods

### Ethics statement

The Medical Ethics Committee of the Erasmus MC approved the study, and all participants gave written informed consent.

### Study population

The Rotterdam Study is a prospective, population-based study, which started with a baseline visit between 1990 and 1993. All inhabitants of a suburb of Rotterdam aged 55 years and over were invited. In total 7983 men and women agreed to participate (response 78%). The rationale and design of the Rotterdam Study have been described elsewhere [24]. Follow-up visits took place in 1993 to 1994 and 1997 to 1999.

From 1997 onwards, participants through 85 years of age completing the third phase of the first cohort were invited to participate in the Rotterdam Coronary Calcification Study and to undergo an electron-beam tomography scan. Of the 3371 eligible, scans were obtained for 2063 subjects (response: 61%). Due to several causes, i.e. metal clips from cardiac surgery, image acquisition data could not be reconstructed or analysed in 50 subjects. Thus, scores were available for 2013 participants. The present study was for several reasons restricted to the asymptomatic population, i.e. subjects without a history of CHD (defined as the presence of myocardial infarction, coronary artery bypass grafting, or percutaneous transluminal coronary angioplasty). The occurrence of myocardial infarction in the past may have led to changes in life-style and medication use to reduce cardiovascular risk. Some of the culprit plaques may also have calcified only after the diagnosis of myocardial infarction. Thus, the resulting calcium score may not be a good reflection of what the calcium score would have been if the participant had not undergone a coronary event. For all these reasons, subjects with a history of CHD (n = 218) were not included in the study. The asymptomatic population consisted of 1795 subjects. We excluded 92 participants (5.1%), because we had too little serum to determine the glomerular filtration rate (GFR). Hence 1703 participants are included in this analysis.

### Cardiovascular Risk Factors

During the third research round a trained research assistant obtained information on smoking habits and medication use from each subject during a home interview. Individuals were classified as never having smoked, a past smoker, or a current smoker. Clinical measurements were obtained during the visit to the research centre. Height and weight were measured, and body mass index was defined as weight in kilograms divided by the square of height in meters. Blood pressure was calculated as the average of 2 consecutive measurements at the right brachial artery with a random zero sphygmomanometer and the participant in sitting position. Hypertension was defined as a systolic blood pressure of ≥140 mm Hg, diastolic blood pressure of ≥90 mm Hg, and/or use of blood pressure–lowering medication for the indication hypertension. After an overnight fast, blood samples were obtained at the research centre. Serum total cholesterol was determined by an automated enzymatic procedure using the Roche CHOD-PAP reagent agent, and HDL was measured with the Roche HDL cholesterol assay using PEG-modified enzymes and dextran sulfate. Glucose was determined enzymatically by the Hexokinase method. Diabetes mellitus was defined as the use of antidiabetic medication and/or a random or postload serum glucose level above 198.2 mg/dL (11.0 mmol/L).

### Assessment of renal function

Serum creatinine was assessed by a nonkinetic alkaline picrate (Jaffe) method [25]. Since the measure of creatinine can vary across different laboratories, we first calibrated our creatinine measures. For this purpose, mean creatinine values from the Rotterdam Study, by sex-specific age groups (<60, 60–69, ≥70 years), were aligned with the corresponding corrected means from the Third National Health and Nutrition Examination Survey (NHANES III) participants [personal communication with Joe Coresh]. The NHANES III creatinine measures were calibrated to the Cleveland Clinic Laboratory, requiring a correction factor of 0.23 mg/dL (20.3 µmol/L) [26]. The glomerular filtration rate (GFR) was estimated by the abbreviated modification of diet in renal disease (MDRD) equation [27] as recommended by the National Kidney Foundation [28]. Estimated GFR (eGFR in ml/min/1.73 m^2^)  = 186×[serum creatinine (in mg/dl)^–1.154^×Age^–0.203^×0.742 (if female)×1.210 (if black)] [29].

### Assessment of Coronary Artery Calcification

Coronary artery calcifications were assessed in the epicardial coronary arteries detected on electron beam tomography scans. Imaging was performed with a C-150 Imatron scanner (GE-Imatron) according to a previously described imaging protocol [30]. Quantification of coronary calcifications was performed with AccuImage software (AccuImage Diagnostics Corp), displaying all pixels with a density >130 Hounsfield units. A calcification was defined as a minimum of 2 adjacent pixels (area = 0.52 mm^2^) with a density >130 Hounsfield units. We placed a region of interest around each high-density lesion in the epicardial coronary arteries. The peak density in Hounsfield Units and the area in mm^2^ of the individual coronary calcifications were calculated. A calcium score was obtained by multiplying each area of interest with a factor indicating peak density within the individual area, as proposed by Agatston et al. [31]. We added the scores for individual calcifications, resulting in a calcium score for the entire epicardial coronary system. Scans were read by 2 readers (R.V., B.S.), the former of whom was at the time a research physician dedicated to coronary calcium research, the latter of whom is an experienced radiologist. Before reading of the scans in our study was started, both readers had a training period in which they read scans and compared calcium scoring results informally. As is known from earlier studies, the interobserver variability for total calcium scores is negligible, also in case of calcium scoring by non-radiologists [32], [33]. The scan readers were blinded to the clinical data of the participants. To conform to the protocol outlines approved by the Medical Ethics Committee, participants were not informed about calcification scores.

### Statistical analysis

Because the distribution of calcium scores was skewed, we used the logarithmic transformation of the calcium scores and added 1 to all calcium scores to deal with values of zero (ln [CAC+1]). Cut-off points for tertiles of GFR were 68.7 and 80.0 mL/min/1.73 m^2^. We used analysis of covariance (ANCOVA) to test for geometric mean differences in CAC between GFR tertiles. We used the highest GFR tertile as the reference group. In linear regression models we tested for interaction between age and GFR, and between sex and GFR. For all analyses we specified two ANCOVA models. In model 1 we adjusted for gender and age. In model 2, we additionally adjusted for the following cardiovascular risk factors: body mass index, hypertension, total cholesterol, HDL cholesterol, smoking and diabetes mellitus. A test for trend was performed with linear regression analysis with the GFR tertiles as ordinal variable. In case of missing values for the cardiovascular variables, values were imputed using the Expectation Maximization method, which is an iterative optimization method to estimate some unknown parameters, given measurement data [34]. In the study population of 1703 participants, information for 1 cardiovascular risk factor was missing for 8%, and information on ≥2 cardiovascular risk factors was missing for 3%. All analyses were performed using SPSS statistical software (version 15.0; SPSS Inc, Chicago, Ill) for Windows.

## Results

Baseline characteristics of the total study population and stratified by median GFR are shown in Table 1. The median age of the group with the lower GFR was 72.1 compared to 68.4 years for the group with the higher GFR. The distribution of different calcium score categories was comparable in both GFR groups. Table 2 shows the distribution of different factors in participants with CAC scores under and above 10.

**Table 1:** Baseline characteristics.

	All subjects (n = 1703)	eGFR <74 3 mL/min/1.73 m^2^ (n = 852)	eGFR >74 3 mL/min/1.73 m^2^ (n = 851)
Age (years)	69.9 (66.0–74.6)	72.1 (67.4–76.1)	68.4 (65.0–72.5)
Sex (male)	731 (42.9)	308 (36.2)	423 (49.7)
Body mass Index (kg/m^2^)	27.0±4.0	27.3±4.0	26.8±3.9
Current smokers	268 (15.7)	103 (12.1)	165 (19.4)
Former smokers	902 (53.0)	458 (53.8)	444 (52.2)
Systolic blood pressure (mm Hg)	143.3±21.0	144.0±20.8	142.7±21.1
Hypertension	1277 (75.0)	664 (77.9)	613 (72.0)
Total Cholesterol (mmol/l)	5.9±0.9	5.9±0.9	5.8±0.9
HDL Cholesterol (mmol/l)	1.4±0.4	1.4±0.4	1.4±0.4
Diabetes mellitus	223 (13.1)	104 (12.2)	119 (14.0)
eGFR (mL/min/1.73 m^2^)	75.2±14.7	64.0±8.3	86.3±10.8
Range eGFR (mL/min/1.73 m^2^)	16.9–164.5	16.9–74.3	74.3–164.5
Coronary artery calcification score	97.5 (9.3–439.4)	108.4 (11.6–446.6)	87.4 (7.7–430.1)
Coronary artery calcification score of 0	171 (10.0)	82 (9.6)	89 (10.5)
Coronary artery calcification score of 1–10	260 (15.3)	117 (13.7)	143 (16.8)
Coronary artery calcification score of 11–99	426 (25.0)	219 (25.7)	207 (24.3)
Coronary artery calcification score of 100–400	397 (23.3)	203 (23.8)	194 (22.8)
Coronary artery calcification score of >400	449 (26.4)	231 (27.1)	218 (25.6)

*Note.* Values are mean ± SD or n (%), except for age and coronary artery calcification the median and interquartile range is given.

**Table 2:** Distribution of different factors in participants with coronary artery calcification scores under and above 10.

	Coronary artery calcification score <10 (n = 431)	Coronary artery calcification score >10 (n = 1272)
Age (years)	68.6±5.1	71.2±5.6
Sex (male)	105 (24.4)	626 (49.2)
Body mass Index (kg/m^2^)	26.5±3.5	27.2±4.1
Current smokers	47 (10.9)	221 (17.4)
Former smokers	195 (45.2)	707 (55.6)
Systolic blood pressure (mm Hg)	138.5±20.5	144.9±20.9
Hypertension	156 (36.2)	1003 (78.9)
Total Cholesterol (mmol/l)	5.9±1.0	5.9±0.9
HDL Cholesterol (mmol/l)	1.5±0.4	1.4±0.4
Diabetes mellitus	36 (8.4)	187 (14.7)
eGFR (mL/min/1.73 m^2^)	75.8±13.3	74.9±15.2
Range eGFR (mL/min/1.73 m^2^)	43.5–126.4	16.9–164.5
eGFR <60 mL/min/1.73 m^2^	41 (9.5)	171 (13.4)
eGFR 60–90 mL/min/1.73 m^2^	165 (38.3)	514 (40.4)
eGFR >90 mL/min/1.73 m^2^	225 (52.2)	587 (46.1)

*Note.* Values are mean ± SD or n (%),

In a linear regression model the interaction term between age and GFR was significant (P = 0.037) indicating that the relation between GFR and CAC might be modified by age. We therefore divided the population in two age categories based on the median age of 69.9 years. Below 70 years cut-off points for tertiles of GFR were 72.9 and 83.5 mL/min/1.73 m^2^. In the age group of at least 70 years these cut-off points were 65.2 and 75.5 mL/min/1.73 m^2^. No significant interaction was found between gender and GFR in a linear regression model (P = 0.142).

**Figure 1:**
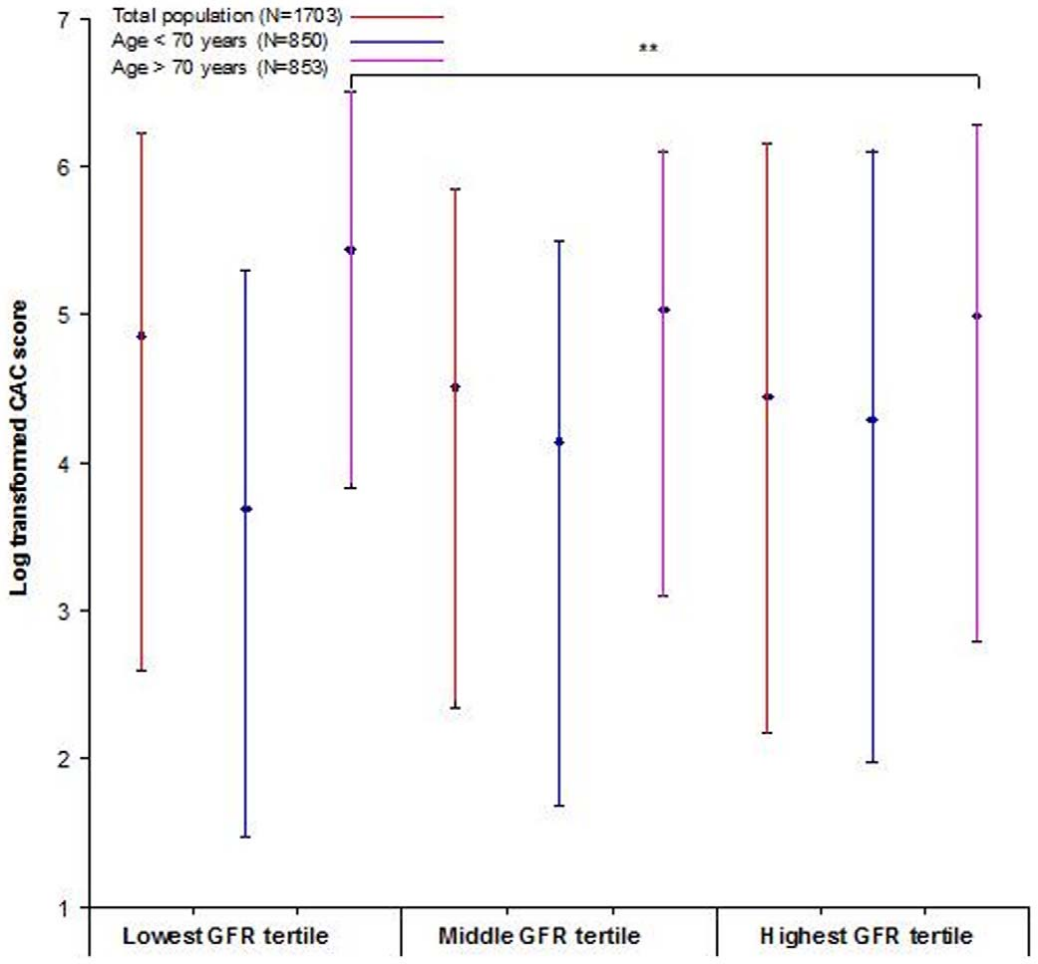
Log transformed CAC score by GFR tertiles in the total population, subject below 70 years and subjects of at least 70 years. Medians (diamonds) and interquartile ranges are presented. ** significant difference between the GFR tertiles after multivariable adjustment (P<0.05)

CAC scores among the GFR tertiles are shown in fig. 1. In the total population, the geometric mean CAC score in the middle and lowest GFR tertiles were not significantly higher compared to the highest GFR category (adjusted for age and gender: geometric mean CAC score 4.1 and 4.3 vs 4.2, P = 0.558 and P = 0.365 respectively; Test for trend P = 0.367). After multivariable adjustment, the geometric mean CAC score in the middle and lowest GFR tertiles also were not significantly higher compared to the highest GFR category (geometric mean CAC score 4.2 and 4.3 vs 4.2, P = 0.996 and P = 0.496 respectively; Test for trend P = 0.497).

Below 70 years CAC scores among the GFR tertiles are shown in fig. 1. The geometric mean CAC score in the middle and lowest GFR tertiles were not significantly higher compared to the highest GFR category (adjusted for age and gender: geometric mean CAC score 3.6 and 3.6 vs 3.9, P = 0.134 and P = 0.121 respectively; Test for trend P = 0.119; multivariable adjustment: geometric mean CAC score 3.7 and 3.6 vs 3.9, P = 0.265 and P = 0.139 respectively; Test for trend P = 0.137)

In the participants who were at least 70 years of age CAC scores among the GFR tertiles are shown in fig. 1. Adjusted for age and gender, the geometric mean CAC score in the lowest GFR tertile was significantly higher compared to the geometric mean CAC score in the highest GFR tertile (geometric mean CAC score 5.0 vs 4.5, P = 0.004. Test for trend P = 0.004). After multivariable adjustment, the lowest GFR tertile also had a significantly higher geometric mean CAC score compared to the highest GFR tertile (geometric mean CAC score 4.9 vs 4.5, P = 0.010; Test for trend P = 0.010).

In a secondary analysis we defined three groups depending on the GFR: group 1 with GFR <60 mL/min/1.73 m^2^ (n = 212), group 2 with GFR between 60 and 75 mL/min/1.73 m^2^ (679) and group 3 with GFR >75 mL/min/1.73 m^2^ (812). No significant differences in mean CAC were found between the three groups in the total population and in the young age group. In the old age group the geometric mean CAC score in group 1 (n = 164) was significantly higher compared to group 3 (n = 295): geometric mean CAC score 5.0 vs 4.5, P = 0.006, test for trend P = 0.005. After multivariable adjustment, group 1 also had a significantly higher geometric mean CAC score compared to group 3 (geometric mean CAC score 4.9 vs 4.5, P = 0.020; Test for trend P = 0.013).

To get further insight in the question whether it is age per se or the low GFR range that is accompanying older age that accounts for the association with CAC, we performed an additional analysis. We matched the GFR ranges of the tertiles in the older age group (these cut-off points were 65.2 and 75.5 mL/min/1.73 m^2^) to the younger age groups and investigated if we could also find a substantial higher CAC in the lowest GFR group in the “young” individuals. Adjusted for age and gender, the geometric mean CAC score of 3.8 in the middle (n = 242) and 3.3 in the lowest GFR group (n = 110) were not significantly different compared to geometric mean CAC score of 3.8 in the highest GFR category (n = 498), P = 0.975 and P = 0.072 respectively. These results suggest that the found relationship between reduced GFR and CAC in the older age group is more likely to be due to older age.

## Discussion

In this population-based study among elderly, we observed that the association between kidney function and CAC depends on age. Under 70 years, the median age, we did not find any significant association of GFR with CAC. Above the median age we did find a significant association of GFR with CAC. In this age group, the geometric mean CAC score in the lowest GFR tertile was significantly higher compared to the highest GFR tertile independent of cardiovascular risk factors.

Several epidemiological studies examined the association between GFR and CAC. Ix et al. [35] evaluated 6749 participants in the Multi-Ethnic Study of Atherosclerosis with a mean GFR of 79 ml/min per 1.73 m^2^ and a mean age of 63 years. In this study kidney function was associated with CAC (relative risk for the prevalence of CAC was 1.41 [95% confidence interval 1.32 to 1.49] for GFR below compared with above 60 m/min per 1.73 m^2^). However, this association was lost after adjustment for age, gender, race, hypertension, and the inflammatory marker IL-6 (relative risk for the prevalence of CAC was 1.03 [95% confidence interval 0.98 to 1.08] for GFR below compared with above 60 m/min per 1.73 m^2^). Fox et al. [11] evaluated 319 participants in the Framingham Heart Study with a mean GFR of 86 ml/min per 1.73 m^2^ and a mean age of 60 years. The authors observed an association between GFR and CAC in unadjusted analysis (spearman correlation coefficient −0.28, P<0.0001), but the association was attenuated after multivariable adjustment for cardiovascular risk factors (spearman correlation coefficient −0.10, P<0.08). Kramer et al. [20] evaluated 2660 participants with a median age of 43.9 years in the Dallas Heart Study. The authors did not observe an association between mild chronic kidney disease (defined as presence of microalbuminuria and GFR >60 ml/min per 1.73 m^2^) and CAC (multivariable adjusted odds ratio for CAC >400 versus CAC ≤10 was 0.97 [95% confidence interval, 0.23 to 4.14] for mild chronic kidney disease compared to no kidney disease). Seyahi et al. [36] evaluated 101 living kidney donors with mean age of 48 years and 99 age- and sex-matched healthy control subjects. The mean GFR was respectively 75.0 and 99.8 mL/min/1.73 m^2^ and both groups were free of diabetes. The prevalence of CAC was not significantly different between kidney donors and controls (13.9% versus 17.2%; P = 0.56). Additionally no significant association was found between GFR and CAC in the kidney donor group (spearman correlation coefficient 0.51, P = 0.62). The above mentioned studies are comparable to our younger age group, both in GFR range and in age. Our study results in the persons below the median age of 70 years are in line with these studies.

The results we found in the older age group are more difficult to compare with previous studies, since the GFR range and age groups hardly match. Studies that found an association of CAC with impaired renal function, comprised relatively young persons on dialysis or with very low GFR ranges [12], [16]–[19]. Kramer et al. [20] did not observe an association between mild chronic kidney disease, but did observe an association between severe chronic kidney disease (defined as GFR <60 ml/min per 1.73 m^2^ excluding end-stage kidney disease) and CAC (multivariable adjusted odds ratio for CAC >400 versus CAC ≤ 10 was 8.35 [95% confidence interval, 1.94 to 35.96] for severe chronic kidney disease compared to no kidney disease).

This study is the first population-based study showing that the association between kidney function and CAC depends on age. Kidney dysfunction is associated with multiple physiological and metabolic changes such as higher tricglycerides, lower high-density lipoprotein levels, higher lipoprotein(a) levels, hypercalcemia, hyperhomocysteinemia, hyperuricemia, elevated serum calcium-phosphorous product, evidence of increased inflammation and oxidative stress, all of which have detrimental cardiovascular effects [37]–[44]. This metabolic milieu may accelerate the CAC process. As renal functional decline slowly advances with older age, renal impairments are probably of longer duration at higher ages and thus above mentioned physiological and metabolic changes could have longer stimulated the CAC process. This could (partly) explain why we did observe an association between CAC and GFR in the old age group, but not in the young age group, even after matching GFR ranges.

### Strengths and limitations

Our study has several strengths, including the large number of participants and standardized information on many potential confounding factors. However, several limitations should be considered. First, creatinine concentration and CAC were measured only once, and thus the reliability of these measurements could not be evaluated. Second, we had no other measures of impaired kidney function available, such cystatin C or albuminuria. Cystatin C may be a better marker of chronic kidney disease than creatinine and may be a better predictor for cardiovascular disease [4]. Third, this study is cross-sectional which makes it unsuitable to draw conclusions about causal inferences. Fourth, in our study, 61% of the invited population participated. Characteristics of the responders were highly similar to those of the nonresponders. There were no significant differences with regard to total and HDL cholesterol levels, hypertension and diabetes mellitus. However, the scanned population was younger (mean age difference, 1.7 years), consisted of relatively more men (46.3% versus 37.8%), was more likely to have a history of smoking (70% versus 63%), had a slightly higher body mass index (27.0 versus 26.7). The only considerable difference was found in the percentage of men and women (8.5% more men among the study population compared with nonresponders). Responders and nonresponders did not show material differences in levels of cardiovascular risk factors. Therefore, we think that other reasons not related to cardiovascular risk may have caused the differential response of men and women. Although we think it is unlikely that selection bias has occurred, we cannot exclude a slight underestimation or overestimation of the studied association. Fifth, in general if the overall analyses show no association between GFR and CAC, then exploration of interaction terms is not appropriate. However, it was a prespecified hypothesis that the association between reduced GFR and CAC might be modified by age as GFR deceases with advanving age [21]–[23]. Sixth, we are the first to have found that age modifies the relation between GFR and CAC. Our results should be confirmed by future research.

### Conclusion

In this population-based study we observed that the association of CAC and GFR is modified by age. In persons under 70 years of age, no association between GFR and CAC was found, while in persons above 70 years the mean CAC in the lowest GFR tertile was significantly higher compared to the highest GFR tertile.
